# Which software packages did researchers use to meta-analyze fMRI data? A literature survey from 2019 to 2024

**DOI:** 10.3389/fnhum.2025.1580808

**Published:** 2025-07-10

**Authors:** Andy Wai Kan Yeung, Natalie Sui Miu Wong

**Affiliations:** ^1^Oral and Maxillofacial Radiology, Applied Oral Sciences and Community Dental Care, Faculty of Dentistry, The University of Hong Kong, Hong Kong, China; ^2^Oral and Maxillofacial Surgery, Faculty of Dentistry, The University of Hong Kong, Hong Kong, China

**Keywords:** coordinate based meta-analysis, GingerALE, SDM, neurosynth, NiMARE

## Abstract

**Introduction:**

There are various methods available for conducting meta-analyses of fMRI data, with coordinate-based meta-analysis (CBMA) being a frequently used approach due to the limited availability of effect size and statistical maps. Since the literature has accumulated many fMRI meta-analyses, several reports have been published to reveal the prevalence of numerous meta-analytic software packages without investigating into the recency of their versions used. To address this gap, a literature survey was conducted to identify the software packages and version numbers used for fMRI meta-analyses published between 2019 and 2024.

**Methods:**

The online databases of Web of Science Core Collection (WOSCC) and Scopus were queried to identify relevant papers. After screening, the analysis consisted of data manually extracted from 820 papers.

**Results:**

The most frequently used software was GingerALE (407 out of 820 papers, 49.6%), followed by SDM-PSI (27.4%) and Neurosynth (11.0%). Overall, 540 papers (65.9%) fully disclosed the names and version numbers of the software packages used in their analyses, whereas 19 papers (2.3%) reported neither the names nor the version numbers. For GingerALE, the most frequently used versions were 2.3.6 and 3.0.2, but it should be noted that versions prior to 2.3.6 have an issue of inflated false positive rates. For SDM-PSI, the most frequently used versions were 5.141, 5.15, 6.21, and 6.22, but the meta-analytic method adopted for version 6 differs from those used in prior versions.

**Discussion:**

To ensure transparency and reproducibility in research, researchers should clearly report the name and version number of software package used.

## Introduction

The fMRI literature has accumulated a number of meta-analytic studies, which used a huge variety of software packages to conduct meta-analyses. FMRI meta-analysis is necessary because individual fMRI studies often suffer from small sample sizes due to the high cost of scanning, the risk of false positives arising from multiple testing and inadequate correction methods, and variability in analysis techniques that can lead to inconsistent results (Button et al., 2013; Yeung et al., 2023).

FMRI meta-analysis can be broadly divided into two approaches: the estimation of effect size versus effect location (Fox et al., 1998). For the former, researchers may extract the effect size reported from individual studies (e.g., percent signal change from a region-of-interest), or other effect measures and process with general meta-analytic software such as Review Manager (RevMan). Since many fMRI studies reported the coordinates of the significantly activated clusters of voxels but not the effect size (Chen et al., 2017), the majority of fMRI meta-analyses focus on the latter, that is effect location, with the mainstream approach called coordinate-based meta-analysis (CBMA), aimed at identifying robust convergence of brain activation across studies. CBMA requires data of the activation foci in the format of brain coordinates, or peak coordinates, with reference to a standard space (Salimi-Khorshidi et al., 2009). Notable CBMA methods included activation likelihood estimation (ALE), commonly performed with the software package called GingerALE (Eickhoff et al., 2009; Eickhoff et al., 2012; Turkeltaub et al., 2012); multilevel kernel density analysis (MKDA), commonly performed with the MKDA toolbox (Wager et al., 2007); and Analysis of Brain Coordinates (ABC), commonly performed with the NeuRoi toolbox (Tench et al., 2017; Tench et al., 2022). It is worth noting that Neurosynth is a website/database that hosts large-scale automated CBMAs resulted from its own methodology, and allows users to type words to generate corresponding CBMAs (Yarkoni et al., 2011). CBMAs have the advantages of good data accessibility (requiring brain coordinates from published studies rather than brain maps), broad literature coverage, data standardization (brain coordinates in Talairach or MNI space), and ease of use; at the same time, they have the disadvantages of data reduction (loss of information), limited spatial precision, and publication bias (Jennings and Van Horn, 2012; Ioannidis et al., 2014; Samartsidis et al., 2017; Chen et al., 2022). On the other hand, if the collected dataset contains information at the voxel-level (e.g., t-maps from statistical parametric mapping, or SPM t-maps), then image-based meta-analysis (IBMA) can be performed. For instance, Seed-based d Mapping with Permutation of Subject Images (SDM-PSI) is a hybrid method that can pool data from studies with only peak coordinates with studies with SPM t-maps, commonly performed with the SDM-PSI toolbox (Albajes-Eizagirre et al., 2019). Please refer to Table 1 for a brief introduction of some representative fMRI meta-analytic software packages.

**TABLE 1:** Brief introduction of some representative fMRI meta-analytic software packages.

Software	Primary methods paper	First version	Latest version	Type of analysis supported	Characteristics
GingerALE	(Eickhoff et al., 2009)	1.0 (13 Apr 2007)	3.0.2 (16 May 2019)	CBMA	Uses the activation likelihood estimation (ALE) algorithm. Runs with GUI.
SDM-PSI	(Radua and Mataix-Cols, 2009)	1.11 (Jul 2009)	6.23 (Feb 2024)	CBMA and IBMA	Uses the seed-based d mapping (SDM) algorithm. Runs with GUI.
Neurosynth	(Yarkoni et al., 2011)	?	?	CBMA	Provides automatic CBMA results after inputting a term, a brain coordinate, etc. Runs with GUI as a website.
MKDA	(Wager et al., 2007)	?	?	CBMA	Uses the multilevel kernel density analysis (MKDA) algorithm. Runs as Matlab scripts.
NeuRoi	(Tench et al., 2017)	?	?	CBMA	Has several choices of algorithms, including analysis of brain coordinates (ABC), and coordinate based meta-analysis of networks (CBMAN). Runs with GUI.
NiMARE	(Salo et al., 2022)	0.0.1 (20 Nov 2019)	0.4.1 (19 Nov 2024)	CBMA and IBMA	Has several choices of kernel-based algorithms for CBMA, including ALE and MKDA; and multiple choices for IBMA, such as Stouffer’s and Fisher’s. SDM is not available. Runs with CLI or API.

Some neuroimaging meta-analytic overviews have been published in the past. For example, (Acar et al., 2024) queried the PubMed database in September 2023 to identify papers that mentioned various meta-analysis methods with the term “meta-analysis” in their title or abstract. Their investigation concluded that ALE was the most frequently used method, with a peak of approximately 90 papers in 2022, to be followed by SDM-PSI and Neurosynth, with their respective peaks at approximately 40 and 30 papers in 2022, whereas few papers mentioned MKDA. There were almost no IBMA papers. Meanwhile, a study published in 2019 analyzed neuroimaging meta-analyses on gustation and found that 20 out of 23 meta-analyses used GingerALE, whereas SDM-PSI and MKDA were each used by one study only (Yeung et al., 2019). Besides, an earlier paper reported that 77% of published neuroimaging meta-analyses indexed in PubMed until 2018 used ALE method, followed by SDM (17%) based on an undisclosed search method (Tahmasian et al., 2019). The dominance of ALE and SDM methods have been reaffirmed by a recent preprint, which reported numbers of 72.7 and 21.2%, respectively, based on an analysis of 899 papers (Oudyk et al., 2025). On a separate note (Tench et al., 2022) mentioned that the most popular CBMA method is probably the ALE algorithm, pointed out that the ALE algorithm has changed over time, and proposed a new method of their own. A research gap exists because Acar et al. (2024), Tahmasian et al. (2019), and Oudyk et al. (2025) reported on the prevalence of methods but not software packages, whereas Yeung et al. (2019) reported the use of software packages for gustatory neuroimaging research only.

This work revisits the topic and provides an update that focuses on the current situation of the fMRI literature. It is important to know not only which software package was used to conduct the fMRI meta-analysis but also its version number to improve transparency and ensure reproducible research (Niso et al., 2022). This is particularly crucial with older versions of GingerALE, which contain bugs that could inflate the false positive rate before version 2.3.6 (Eickhoff et al., 2017). A software package may have a long history with multiple versions, where some updates provide minor enhancements or additional functionality, while others fix major errors or bugs, making it important for users to be aware of these changes especially the latter ones. For example, readers may refer to (Yeung, 2024) for a timeline that illustrates the various versions of GingerALE that introduced and fixed major errors. Field experts have made efforts to consolidate CBMA and IBMA methods with a simple and shared interface to reduce brand loyalty to any particular algorithm and to encourage between-method comparisons, one of which is called Neuroimaging Meta-Analysis Research Environment (NiMARE) with its initial release in November 2019 (Salo et al., 2022). Hence, it would be interesting to know whether NiMARE has had an increasing publication share over the past few years.

Given this context, the purpose of this literature survey is to identify what software packages and their version numbers that have been used for fMRI meta-analyses published from 2019 to 2024, inclusive.

## Methods

**FIGURE 1:**
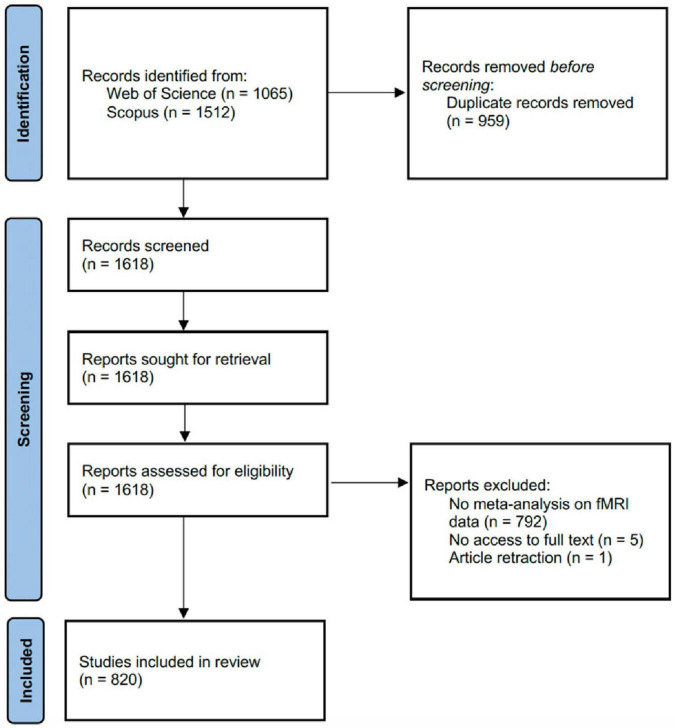
Screening process of the fMRI meta-analysis literature.

The online databases of Web of Science Core Collection (WOSCC) and Scopus were queried on 9 May 2025. The title, abstract and keyword fields were searched with the following search string: (fMRI OR “functional magnetic resonance imaging” OR “functional MRI”) AND (meta-analy*). Papers were only included if they were labeled as article or review, written in English, and published during 2019–2024. The search yielded 1,065 papers from WOSCC, and 1,512 papers from Scopus. The records of the papers were compiled into a list and passed into Microsoft Excel for de-duplication with reference to the Digital Object Identifiers (DOIs) and paper titles. After de-duplication, 1,618 papers remained. Among these 1,618 papers, 792 were excluded due to no meta-analysis on fMRI data after accessing their full text, 5 were excluded due to no access to the full text, and 1 was excluded due to article retraction. For the remaining 820 papers that entered the analysis, the name and version number of the software packages used to perform fMRI meta-analysis were manually extracted, if present (Figure 1). Both authors independently conducted the paper screening and data extraction. Any disagreements were resolved through discussion to reach a mutual consensus.

## Results

**FIGURE 2:**
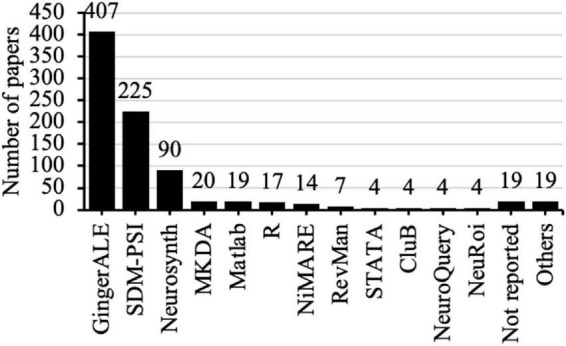
Frequency count of different software packages used to perform fMRI meta-analysis. Out of the 820 papers that performed fMRI meta-analysis, some used more than one software. Therefore, the total frequency count amounted to 853.

The coded data sheet was provided as the Supplementary File 1. Among the 820 papers that conducted fMRI meta-analyses, some used multiple software programs. As a result, the total count of software usage reached 853. The most frequently used software was GingerALE (407 out of 820 papers, 49.6%), followed by SDM-PSI (27.4%) and Neurosynth (11.0%) (Figure 2).

Meanwhile, Table 2 shows the frequency counts per year. The temporal trends of the 3 software packages with the highest publication share are as follows. The annual publication share of GingerALE was always slightly over one-half until 2023, when it dropped to 47.4% and further to 42.6% in 2024. The publication share of SDM-PSI increased every year from 20.8% in 2019 to 32.3% in 2024. Neurosynth had approximately 10% publication share per year during 2019–2024.

**TABLE 2:** Annual frequency count of different software packages used to perform fMRI meta-analysis.

Software	2019	2020	2021	2022	2023	2024	Total
GingerALE	55 (54.5%)	58 (51.8%)	72 (51.8%)	83 (52.2%)	73 (47.4%)	66 (42.6%)	407 (49.6%)
SDM-PSI	21 (20.8%)	25 (22.3%)	33 (23.7%)	47 (29.6%)	49 (31.8%)	50 (32.3%)	225 (27.4%)
Neurosynth	8 (7.9%)	18 (16.1%)	14 (10.1%)	17 (10.7%)	15 (9.7%)	18 (11.6%)	90 (11.0%)
MKDA	5 (5.0%)	4 (3.6%)	2 (1.4%)	5 (3.1%)	2 (1.3%)	2 (1.3%)	20 (2.4%)
Matlab	5 (5.0%)	3 (2.7%)	5 (3.6%)	2 (1.3%)	2 (1.3%)	2 (1.3%)	19 (2.3%)
R	0	1 (0.9%)	6 (4.3%)	0	4 (2.6%)	6 (3.9%)	17 (2.1%)
NiMARE	0	0	4 (2.9%)	1 (0.6%)	4 (2.6%)	5 (3.2%)	14 (1.7%)
RevMan	0	0	1 (0.7%)	1 (0.6%)	1 (0.6%)	4 (2.6%)	7 (0.9%)
STATA	0	0	0	0	1 (0.6%)	3 (1.9%)	4 (0.5%)
CluB	1 (1.0%)	0	0	1 (0.6%)	0	2 (1.3%)	4 (0.5%)
NeuroQuery	0	0	0	1 (0.6%)	1 (0.6%)	2 (1.3%)	4 (0.5%)
NeuRoi	0	1 (0.9%)	0	2 (1.3%)	0	1 (0.6%)	4 (0.5%)
Not reported	6 (5.9%)	3 (2.7%)	4 (2.9%)	3 (1.9%)	1 (0.6%)	2 (1.3%)	19 (2.3%)
Others	7 (6.9%)	2 (1.8%)	2 (1.4%)	1 (0.6%)	6 (3.9%)	4 (2.6%)	22 (2.7%)
Annual paper count	101	112	139	159	154	155	820

Out of the 820 papers that performed fMRI meta-analysis, some used more than one software. Therefore, the sum of percentages in each year could be > 100%.

Overall, 540 papers (65.9%) fully disclosed the names and version numbers of the software packages used in their analyses (Table 3). In contrast, 19 papers (2.3%) reported neither the names nor the version numbers of the software packages used. The most frequently used versions for GingerALE were 2.3.6 and 3.0.2, whereas the most frequently used versions for SDM-PSI were 5.141, 5.15, 6.21, and 6.22 (Tables 4, 5).

**TABLE 3:** Breakdown of the disclosure of the names and version numbers of software packages used in the analyzed papers.

Condition	Frequency (% of 820)
Reported all names and version numbers	540 (65.9%)
Reported all names but no version number	242 (29.5%)
Reported all names but missed some version numbers	19 (2.3%)
No name and no version number	19 (2.3%)
Total	820

**TABLE 4:** Annual publication counts of different versions of GingerALE.

Year	Version number of release date											Annual sum
		11-Jan-13	30-Sep-14	7-May-14	19-Nov-15	26-Apr-16		5-Feb-19	2-May-19	16-May-19		
	**?**	**2.3**	**2.3.1**	**2.3.2**	**2.3.5**	**2.3.6**	2.3.7*	**3.0**	**3.0.1**	**3.0.2**	3.0.3*	
2019	3	3	1	2	0	42	1	1	0	2	0	55
2020	7	0	0	0	1	25	0	6	0	19	0	58
2021	6	2	0	2	0	12	0	1	0	48	1	72
2022	12	1	0	0	0	13	0	1	0	56	0	83
2023	3	1	0	0	0	7	0	2	1	59	0	73
2024	5	2	0	0	0	5	0	0	0	54	0	66

*Versions 2.3.7 and 3.0.3 are not officially documented. Release dates were retrieved from https://brainmap.org/ale/readme.html (accessed on 16 May 2025).

**TABLE 5:** Annual publication counts of different versions of SDM-PSI.

Year	Version number of release date														Annual sum
		May-15			Aug-16	Oct-16	Dec-16		Apr-18	Apr-19	Jul-19	Nov-19		Feb-24	
	**?**	**4.31**	5*	5.1*	**5.12**	**5.14**	**5.141**	5.142*	**5.15**	**6.11**	**6.12**	**6.21**	6.22*	**6.23**	
2019	10	2	0	0	1	0	5	1	2	0	0	0	0	0	21
2020	9	2	0	0	0	2	5	1	3	1	0	2	0	0	25
2021	10	0	0	1	1	0	0	0	11	0	1	9	0	0	33
2022	15	0	1	0	0	0	1	0	14	0	1	13	2	0	47
2023	8	0	0	0	0	0	0	0	19	0	0	20	2	0	49
2024	18	0	0	1	0	0	1	0	12	0	0	9	7	2	50

*Versions with unclear release dates. Release dates were retrieved from the Readme file after downloading the software from https://www.sdmproject.com/software/ (accessed on 16 May 2025).

**FIGURE 3:**
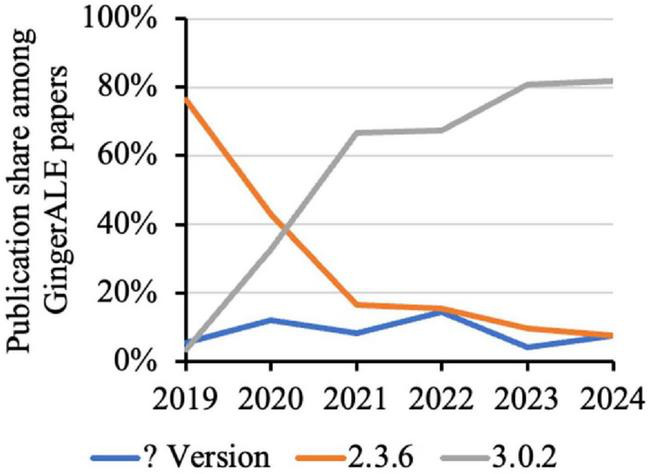
Change in the annual publication share of commonly used versions of GingerALE. “? Version” refers to papers that did not report the exact version number. The publication share is computed from GingerALE papers, not all meta-analysis papers.

Among GingerALE papers, the publication share of version 2.3.6 (released on 26 April 2016) decreased from 76.4% in 2019 to 7.6% in 2024, as the share of version 3.0.2 (released on 16 May 2019) increased from 3.6% in 2019 to 81.8% in 2024 (Figure 3). Their changes were most prominent from 2019 to 2021, and became more mild since then. It is worth noting that two versions of GingerALE, namely versions 2.3.7 and 3.0.3, were each reported in one paper, but they are not officially documented. Furthermore, 15 papers (3.7% of 407) used older versions of GingerALE (prior to 2.3.6), which suggests a higher risk of false positive results.

**FIGURE 4:**
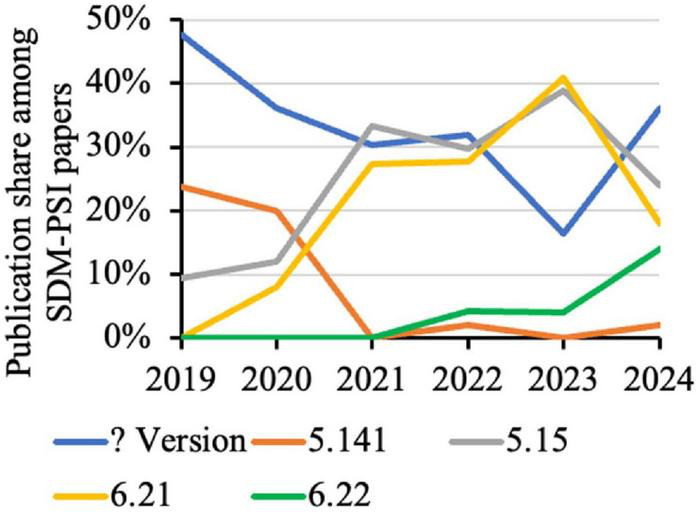
Change in the annual publication share of commonly used versions of SDM-PSI. “? Version” refers to papers that did not report the exact version number. The publication share is computed from SDM-PSI papers, not all meta-analysis papers.

Among SDM-PSI papers, version 5.141 (released in December 2016) was the dominant version in 2019 and 2020 (at least 20%), but the publication shares of versions 5.15 (released in April 2018) and 6.21 (released in November 2019) gradually increased and peaked at 38.8 and 40.8% in 2023, respectively (Figure 4). The publication share of versions 5.15 and 6.21 showed clear decline from 2023 to 2024, during which the share of version 6.22 showed a sharp increase from 4.1 to 14.0%. The ratio of SDM-PSI papers that did not report the version number dropped from 47.6% in 2019 to 36.0% in 2024.

## Discussion

This literature survey has confirmed that GingerALE was the most frequently used software package for fMRI meta-analysis whereas the publication share of SDM-PSI has continued to increase from 2019 to 2024. At the same time, more and more papers have used other software packages such as Neurosynth and NiMARE. The fMRI meta-analytic literature has been dominated by these 2 CBMA meta-analytic software packages. Foundational work by Turkeltaub et al. (2002) and Chein et al. (2002), summarized in Laird et al. (2005), established the methodological framework for ALE, emphasizing their ability to integrate coordinate-based data for cross-study comparisons. After testing convergence of activation across studies, methodological advancements have enabled meta-analytic connectivity modeling (MACM) (Robinson et al., 2010) and co-activation-based parcellation (CBP) (Eickhoff et al., 2011). It is unclear why the prevalence of SDM-PSI was on the rise over the last few years, but it does have advantages over ALE in terms of effect size integration and handling of both positive and negative findings (Albajes-Eizagirre and Radua, 2018; Albajes-Eizagirre et al., 2019). However, no method is perfect. The dominance of one or two methods may reduce the incentive for researchers to develop alternative methods, and novice users may be more hesitant to try these alternatives.

A minority of papers that used GingerALE have either reported the use of older versions (prior to 2.3.6) or not reported the version number. This is not a good phenomenon, as the developer team of GingerALE has already released a debugged version in 2016 that fixed some major issues with inflated false positive rate, with a clear documentation published in 2017 (Eickhoff et al., 2017). As the surveyed meta-analytic papers were all published since 2019, ideally all of them should be using newer versions of GingerALE that have been debugged. The effects on the results could be potentially huge. For instance, an fMRI meta-analysis on perceptual decision making that used GingerALE version 2.3 originally reported 10 clusters with significant activation for a contrast of task > control (Keuken et al., 2014). After re-analyzing data with a debugged version of 2.3.6, all 10 clusters became non-significant (Keuken et al., 2017). Other re-analyses showed less dramatic changes to the initial conclusions, such as a reduction from 36 significant clusters (Belyk et al., 2015) to 4 (Belyk et al., 2017), from 12 significant clusters (Garrigan et al., 2016) to 8 (Garrigan et al., 2016), and an exclusion of three previously significant brain regions (unclear number of significant clusters) (Smith et al., 2016; Smith and Delgado, 2017). Readers should be aware that the examples listed here may be affected by publication bias. It could be possible that many re-analyses showing no significant changes from the original findings ended up as unpublished findings.

Though the issue of inflated false positive rate has not been reported for SDM-PSI, it is still advisable to report version number and use most updated version of the software package whenever possible due to a different issue. A major change of SDM-PSI from version 5 to version 6 was the adoption of permutation so that it tested whether effects were non-null in a given voxel, but no longer tested whether findings across studies tended to converge around it (Albajes-Eizagirre and Radua, 2018; Albajes-Eizagirre et al., 2019). According to the Readme document of SDM-PSI version 6.23, the first officially documented version 6 of SDM-PSI was version 6.11 released in April 2019. Ideally, most of the surveyed meta-analysis papers should have used version 6.11 or newer versions, except those papers published in early 2019. It was largely unclear why the publication share of version 5.15 (released in April 2018) continued to increase year by year and nearly matched the publication share of version 6.21 (released in November 2019). In 2024, though, both versions declined whereas versions 6.22 and 6.23 began to get some publication share. Comparing meta-analytic results between studies using the old (version 5 or older) and new versions (version 6) of SDM-PSI may be conceptually inaccurate due to the fundamentally different methodology. Though recent guidelines for conducting and reporting neuroimaging meta-analysis did not explicitly recommend researchers to list the exact software version number during reporting (Müller et al., 2018; Tahmasian et al., 2019; Manuello et al., 2022), this general practice is highly recommended in reporting an fMRI study (Poldrack et al., 2008).

On a separate note, not many studies in the dataset used NiMARE to conduct fMRI meta-analysis. The concept behind NiMARE is very good: An environment/ecosystem that allows users to conduct multiple types of meta-analyses based on a number of algorithms available from the literature and interact with online databases of brain coordinates and fMRI images. However, since NiMARE runs on Python and is operated through either command line interface (CLI) or application programing interface (API), some researchers may prefer to use existing toolboxes or software packages with a guided user interface (GUI) that allow for operation by pressing buttons, rather than typing computer code. Specifically, those who possess extensive expertise in the domains being meta-analyzed, such as physiology, psychology, or pathology, may not have the programing skills necessary to efficiently utilize CLI or API. This is particularly relevant given the interdisciplinary nature of neuroimaging research, where specialists from diverse backgrounds collaborate. The preference for GUIs is evident in the continued use of software packages such as GingerALE and SDM-PSI. However, analyses with CLI may have better provenance and reproducibility than with GUI, as users may press buttons wrongly and there is usually no action log. Some surveyed studies in the dataset opted to use both GingerALE and SDM-PSI to conduct CBMAs and compare results instead of performing them “centrally” through NiMARE. Hence, NiMARE and similar solutions could consider to build a simple GUI besides command line interface, to attract users from a more diverse background.

This study has some limitations. First, the analysis was restricted to papers published in a short period. The current findings may not be readily applied to the past literature. Second, some papers not indexed by WOSCC or Scopus might be omitted by this study, though they might only account for a small share of the literature. Third, manual data extraction is labor demanding and may be prone to human mistakes. The future development of text scraper with artificial intelligence and availability of literature full texts with open access should enable a replication of this study in a much larger-scale. Future studies can also consider more advanced analyses, such as to reveal any relationship between the impact factors of the journals and software selection.

## Conclusion

Based on a literature survey of papers published during 2019–2024, GingerALE was the most frequently used software package for conducting fMRI meta-analysis, followed by SDM-PSI and Neurosynth. The issue of not reporting the version number of the software package was more serious for SDM-PSI than GingerALE, but the situation has been improving quickly for the former. Another issue for using SDM-PSI was the continued use of its old version together with its latest version. Unlike the old versions, its latest version (version 6) uses permutation to test whether effects were non-null in a given voxel, instead of whether findings across studies tended to converge around it. The methodologies to meta-analyze fMRI data are diverse. Researchers should clearly report the details including the name and version number of software packages used, so that readers can better understand what has been done and how results could be compared across studies.

## Data Availability

The original contributions presented in the study are included in the article/Supplementary material, further inquiries can be directed to the corresponding authors.
